# Effect of Phenol Application Time in the Treatment of Onychocryptosis: A Randomized Double-Blind Clinical Trial

**DOI:** 10.3390/ijerph181910478

**Published:** 2021-10-06

**Authors:** Juan Manuel Muriel-Sánchez, Manuel Coheña-Jiménez, Pedro Montaño-Jiménez

**Affiliations:** Departamento de Podología, Facultad de Enfermería, Fisioterapia y Podología, Universidad de Sevilla, Calle Avicena s/n, 41009 Sevilla, Spain; murielsanchezjm@gmail.com (J.M.M.-S.); mcohena@us.es (M.C.-J.)

**Keywords:** foot pathologies, onychocryptosis, phenol, ingrown toenail, chemical matricectomy

## Abstract

Background: In the treatment of Onychocryptosis, chemical matricectomy with 88% phenol solution is one of the most common surgical procedures due to a recurrence rate of less than 5%, but it may produce a delay in healing time. The objective was to compare the healing time between phenol applications of 30 or 60 s. Methods: A comparative, prospective, parallel, randomized, and blinded clinical trial was registered with the European Clinical Trials Database. Twenty-seven patients (54 feet) with 108 affected nail folds were randomized and treated with chemical matricectomy with phenol. Each hallux was randomly assigned to one of two groups (60 vs. 30 s phenolization). Each patient and one investigator were blinded to the phenol application time in each foot. The outcome measurements were healing time, recurrence, pain, post-surgical bleeding, inflammation, and infection rate. Results: The 30 s application presents a shorter healing time (14.93 ± 2.81 days vs. 22.07 ± 3.16 days; *p* < 0.001) with a similar recurrence rate (*p* = 0.99). Post-operatory bleeding, pain, inflammation, and the infection rate did not show significant differences (*p* > 0.05). Conclusions: The 30 s phenol application time offers a shorter healing time than 60 s without affecting the effectiveness of the procedure, showing the same rate of complications.

## 1. Introduction

Ingrown toenail is a pathologic condition of the nail apparatus in which the nail plate damages the nail fold. This condition causes inflammation, pain, and functional limitation [1]. To treat the ingrown toenail there are several surgical procedures describes in the literature [1,2,3,4,5], as well as its risk factors and etiology [6]. 

Matricectomy applying 88% phenol solution, a non-incisional surgical option, is one of the most common surgical procedures for the definitive treatment of onychocryptosis. It was first described by Boll in 1945, and it refers to the application of phenol after a curettage of the matrix tissue [7]. It has shown very high effectiveness rates and a very low recurrence rate, but it presents a long healing time (2–4 weeks). In 1956, Nyman introduced the application of alcohol after cauterization to neutralize the action of phenol [8].

Subsequent studies determined the amount and concentration of alcohol needed [9]. Numerous modifications have emerged from it to reduce healing time without affecting the effectiveness of the procedure [10,11]. The most relevant modifications are in terms of the phenol application time, phenol concentration, and removal of the cauterized tissue [2].

Boll made a 30 s application of phenol [7]. In 1953, Gottlieb modified the application times, making two 30-s applications [12]. In 1956, Nyman made an application of 30 s and one of 40 s [8]. Six years later, Suppan and Ritchlin made two applications of phenol, two and three minutes, respectively [13]. It is true that there is a histological study on cadavers, which states that it takes 4 min to completely necrotize the matrix tissue [14]. After the study conducted by Boberg et al. (2002), the concentration of the phenol and the minimum application time must be 89% and 1 min for necrosis to occur throughout the thickness of the epithelium [15]. 

Recently, in a study to verify the healing time reduction of curettage after segmental phenolization, 1-min phenolizations were performed, and it was observed that the time of application of the phenol did not influence the effectiveness of the technique [2]. 

The main aim of this research was to find out the effect of phenol application time on healing time process. The secondary objective was to value the effect that both application times had on recurrence rate, pain, inflammation, infection rate, post-surgical bleeding, and infection rate.

## 2. Materials and Methods

### 2.1. Design

A double-blind clinical trial was carried out according to the Consolidated Standards of Reporting Trials (CONSORT) guidelines [16]. The participants were recruited from Podiatric Clinical Area of the University of Seville (Spain) during 2019. Participating patients gave written consent and follow up was for 12 months. 40 patients were recruited and a total of 27 patients were enrolled with a medial and lateral onychocryptosis in both hallux, and all of them received previously conservative treatment, i.e., removal of the partial ingrown nail plate, without resolution of the pathological condition. 

The clinical trial was registered in the Australian New Zealand Clinical Trials Registry (trial id: ACTRN12619001719123) and the European Clinical Trial Database (EudraCT id: 2019-002219-24). The research was also approved by the Bioethics Committee of the Government of Andalusia. Further, we followed ethical standards guidelines for research and experimentation in human participants reported in the Declaration of Helsinki at the 64th World Medical Assembly (Fortaleza, Brazil). 

### 2.2. Participants

**Figure 1:**
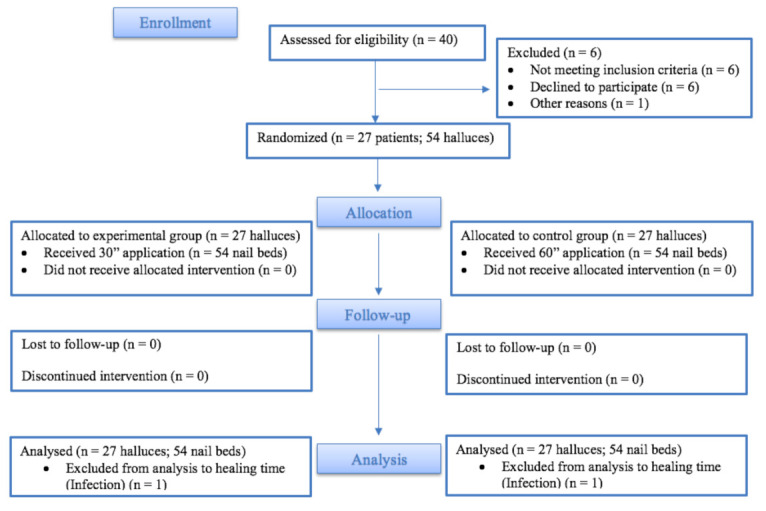
CONSORT flow diagram.

The assignation of participants to control or experimental group was randomized using Microsoft Excel choosing “random function between” to determine which nail matrix receives a 30 s phenol application and which nail bed of the other hallux receives a 60 s phenol application (Figure 1). The same researcher performed the randomization, enrolled of participants, and assigned the time of intervention to halluces. Each patient and the independent outcome measurement researcher were blinded to the phenol application time assigned to each hallux to each nail bed side. The patient cannot see the procedure because the surgery is performed with a censor.

The inclusion criteria were bilateral onychocryptosis in both hallux in stages I or IIa [1], older than 18 years, not having bone pathology confirmed by X-rays, an indication of chemical partial matricectomy. The exclusion criteria were to have chronic illnesses, circulatory problems, labile diabetes, pregnancy, having wound healing disorders, allergy to phenol, or previous ingrown nail surgery. The primary outcome parameter was healing time. The secondary outcomes were recurrence rate, inflammation, post-operatory pain, bleeding, and infection.

### 2.3. Surgical Procedure

Using 2% mepivacaine, a digital block of each hallux was performed; preoperative surgical antisepsis was performed at both feet, and for local hemostasis a digital tourniquet was applied. Using a free elevator, the medial and lateral nail plate was separated from the nail bed and the eponychium and were removed.

**Figure 2:**
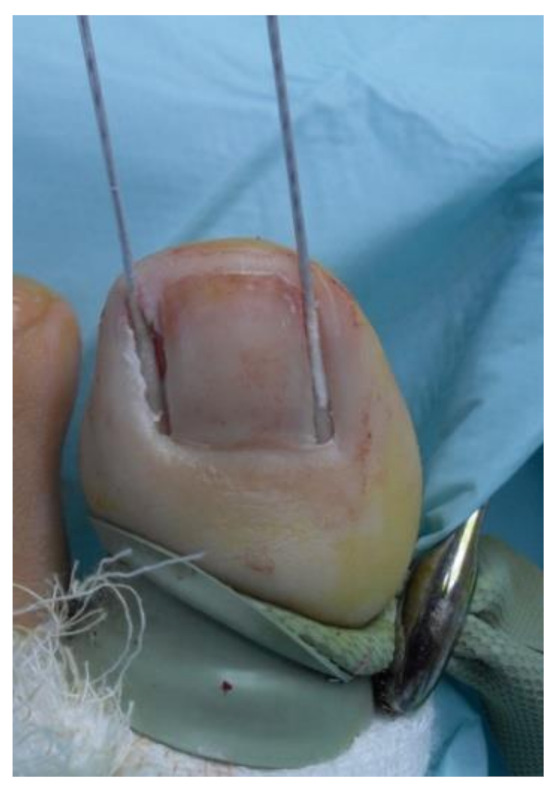
Cotton Swab 88% phenol soaked applied at both sides of matrix and the nail bed for 1-min at control group.

Afterwards, a cotton swab was soaked in 88% phenol and then applied at both nail matrix and nail bed of hallux for 1 min at control group (Figure 2), and for 30 s at the same places of contralateral hallux as the experimental group. 

The matrix and nail bed were irrigated with a sterile 0.9% saline solution for one minute. The surgical wounds were treated with sulfadiazine silver cream and non-stick absorbent sterile polypropylene and covered with sterile gauze and bandage.

### 2.4. Outcome Measurements

The same clinician generated the randomization and performed the surgical procedures in all patients. Another blinded independent clinician, who did not participate in the study, assessed the variables with photographic and clinical follow-up. Patients were seen at 72 h and twice a week until healed. The follow up was completed with daily photographs. A surgical checklist was used for patients’ safety [17].

**Figure 3:**
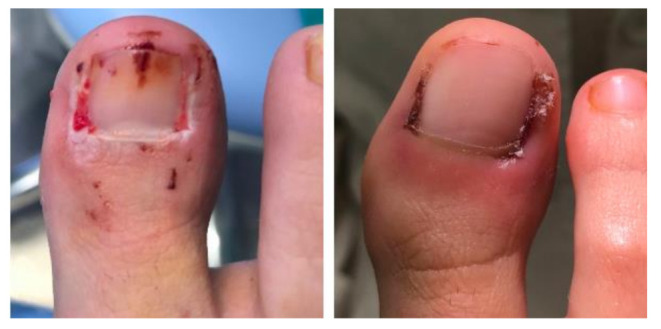
Appearance of the wound at 13 days post op. To the left, granulation tissue is still visible (control group; 60 s). On the right, the granulation tissue has disappeared (experimental group; 30 s).

The primary outcome measurements were healing time. The healing time was measured as previously described criteria [2,18,19], considering the period of time between ending surgical procedure and resolution of the postoperative period. These criteria were absence of exudate at gauze; formation of scab covering the wound; the wound must be kept uncovered; no signs of infection or inflammation at nail folds; no signs of erythema or hypergranulation tissue (Figure 3). 

The secondary outcome measurements were recurrence, post-surgical bleeding, pain, infection, and inflammation. To measure recurrence, a follow-up of a minimum of 6 months was considered [20]. On the other hand, the growth of asymptomatic nail spicule was considered a sequel and not a recurrence [21]. 

The bleeding was achieved from the photographic assessment during the first dressing and classified as light, moderate or abundant by the independent clinician. The bleeding was mild when this partially stains the dressing and gauze, moderate when totally stains the dressing and partially the gauze, and abundant when it stains dressing and much of the gauze and is still bleeding [2]. A 10-point visual analogical scale (VAS) was used. Pain was assessed 24, 48, and 72 h after surgery by VAS, being 0 = no pain and 10 = maximum pain imaginable. The patients were asked to rate their pain by choosing a value on the VAS scale [22]. Inflammation was assessed measuring circumference by flexible ruler (Devon Industries 1-800, Inc., Devon, PA, USA) at proximal nail fold before surgery and during follow up [2,11]. Infection was determined when there was drainage, erythema, and pain [23].

### 2.5. Sample Size Calculation

The sample size required for the research was calculated using CTM-1.1 (Glaxo Wellcome SA, Madrid, Spain). The primary outcome was to detect a clinically relevant difference of 6 days in mean healing time between the groups, so considering a two-tailed test, with an α error of 5%, an β error of 20%, with a statistical power of 80%, and estimating a follow-up loss rate of 10%, a minimum participant in each group was 13 halluces.

### 2.6. Statistical Analysis

The categorical variables were expressed by frequencies and percentages; the quantitative variables in median and standard deviations, and 95% confidence interval (CI), median, and interquartile range. The Shapiro–Wilk was applied to test the normal distribution of variables (*p* > 0.05). Independent t student test was used for parametric data, and Mann–Whitney U test was used for non-parametric data. Chi-square test with Yates continuity correction was applied to the categorical variables. SPSS 22.0. software (SPSS, Inc., Chicago, IL, USA) for statistical analysis was used, and statistically significant differences were set at *p* < 0.05 with a 95% CI.

## 3. Results

### 3.1. Descriptive Data

A total of 27 patients, 19 women and 8 men, and 54 feet and 108 nail folds were registered performed during 2019. The average age of the sample was 36.0 years (S.D. = 10.7). In addition, 70.4% (n = 76) belonged to women and 29.6% (n = 32) to men. A total of 54 nail beds were treated applying 30” and another 54 nail beds with 60” of 88% phenol in experimental and control group, respectively. All variables except BMI, Height, and Age showed a normal distribution (*p* < 0.05).

### 3.2. Outcome Measurements

The clinical results are showed in Table 1. There were significant differences (*p* < 0.05), with healing time being almost 7 days lower for the experimental group. The other variables measured did not show significant differences between groups.

## 4. Discussion

The objective was to find out the effect of phenol application time on the healing time process. In partial chemical matricectomy, the phenol effect is to denature proteins, followed by cell death [2]. A long healing time is one of the main disadvantages of chemical matricectomy. Several studies have examined the healing time after partial phenolization. The results are presented in Table 2. The results that the researchers report are inconsistent, as the healing criteria used are not homogeneous. In the present study, significant differences were found in healing time between the groups in favor of the experimental group.

One study conducted by Boberg et al. (2002) reported the concentration of the phenol and the minimum application time must be 89% and 1-min of application, since from these parameters it has been proven, after a microscopic analysis, which occurs necrosis throughout the thickness of the epithelium [15]. We used 88% phenol solution in 60 and 30 s applications, respectively.

In our study, the healing time was 22.1 ± 3.2 days for the control group and 14.9 ± 2.8 days for the experimental group. Granulation tissue formation and resolution was faster in the experimental group. It was treated with antiseptics until healed. Van der Ham et al. (1990) reported a similar healing time that was obtained in the present study (15.4 ± 4 days) using a 3-min application of 80% phenol, but without specifying the healing criteria used [24]. A retrospective study showed a healing time from 2 to 4 weeks [25], with similar results to a prospective research that reported the return to normal activity after partial chemical matricectomy in 3.89 weeks [26]. Other authors have reported healing times of 14 to 18 days using 90% phenol but with much longer application times (8–10 min) [27].

A study designed to determine the efficacy and safety of the chemical matricectomy compared different application times with Phenol (89%) was applied for 1, 2, or 3 min and the best results with regard to postoperative complications were obtained in the group to which phenol was applied for 1-min, with a healing time of 13.5 ± 3.9 days; for 2 and 3 min were 17.5 ± 2.8 days and 17.1 ± 2.6 days, respectively [28]. This healing time was longer than our results. Hassel et al. (2010) reported, with a phenol application of 2-min, a healing time of 7 days, whose criterion was the relief of symptoms and the capacity to recuperate daily life activities [29]. They considered a healed nail when the patients were able to walk and wear closed shoes. This is usually no issue after phenolization, but is a main advantage compared to most types of surgery.

Vaccari et al. (2010) obtained a healing time of 14–28 days in partial phenolization with three applications of 1-min [30]. Álvarez et al. (2012) performed a study to examine the effect of curettage on healing time, and they obtained that curettage after segmental phenolization reduced healing time [2]. Recently, a clinical trial, the aim of which was to compare the chemical matricectomy with phenol and the aesthetic reconstruction, reported a healing time of 21.3 days using a 88% phenol solution with 60 s application [19].

Although studies did not exist that are similar to this one, in order to be able to establish complete comparisons, our results demonstrated a high effectiveness with both phenol application times. We have considered effectiveness as being the assessable recurrence rate 6 months after surgery [23], and our results were 3.6% in the experimental group and 1.8% in the control group. These results agree with another study obtained recurrence rates of 4.5% after the partial phenolization [31]. Hassel et al. (2010) reported a recurrence rate of 31.5% for the chemical matricectomy with phenol [29]. These results were contrary to those reported by Morkane et al. (1984), they obtained a low recurrence rate [32].

Álvarez et al. (2012), in a randomized clinical trial, obtained a recurrence rate of 0% to partial matricectomy with phenol during 60 s [2]. These results agree with Karaka and Dereli (2012) that reported 99.7% of effectiveness with a 24-month follow-up period, with a phenol application of 2-min [33]. In line with this, Pérez-Rey et al. (2014) obtained a recurrence rate of 1.1% for NaOH chemical matricectomy during 30 s [34]. Recently, a clinical trial has reported 2.8% to chemical matricectomy with a 1-min phenol application [19].

Only a few studies have considered bleeding after partial matricectomy with phenol. Some of them have shown phenolization to reduce bleeding because of the hemostatic effect attributed to phenol [2,32]. It must be taken into account that the procedures were carried out under conditions of ischemia. In line with another clinical trial, we did not obtain significant differences for the bleeding indicator [19], differing from other studies where the bleeding was greater after removing the cauterized tissue with a curettage [2]. Recently, other authors have reported platelet-rich fibrin, rather than nitrofurazone, in conjunction with chemical matricectomy performed with 1-min of 88% phenol solution reduced bleeding after nail surgery [11].

Some authors have suggested that phenol may reduce postoperative pain as a result of neurolytic effect, but only a few studies have analyzed this variable using pain scales [2,19,22,29]. Post-surgical pain was greater after 24/48/72 h for the experimental group. However, statistically significant differences between the two were not shown. The surgical wounds were treated with sulfadiazine silver cream. Recently, a clinical trial reported that Platelet-Rich Fibrin reduced the wound cicatrization period and bleeding after nail surgery [11]. Some authors argue that infection of the wound as a consequence of bacterial infection may cause the delay in healing after partial matricectomy with phenol, and they advocate for systematic use of preoperative antibiotic prophylaxis [2,35]. Evidence does not support use of preoperative antibiotic prophylaxis in onychocryptosis surgery, except in special patients with infective onychocryptosis [2].

Our research presented some limitations, as the assessment of pain by patient due to pain were in the same hallux, and patients sometimes were not able to discriminate if the pain came from medial or lateral matricectomy of the same hallux. This fact could be resolved by designing a study evaluating only one nail fold in each hallux. The study is carried out in a single center, a multicenter study would be more representative for the research variables. Thus, our results are to be treated with caution and further studies more representative of the general population are needed. A longer follow-up period would improve the strength of these results.

### Implications for Clinical Practice

Reducing the healing time process can lead to a return to normal activity of patients early, this being beneficial for society. It is a clinical trial from practical onychocryptosis problem solving. Clinical dermatological practice is more beneficial for the patient and the clinician. The findings can help the clinician maximize the benefits and limit the detrimental effects of the matricectomy with phenol.

## 5. Conclusions

The 30 s phenol application time offers a shorter healing time than 60 s without affecting the effectiveness of the procedure, showing the same rate of complications.

## Figures and Tables

**Table 1:** Clinical variables studied at control and experimental groups.

Outcome Measurements	Control Group (60 s) (n = 54 Nail Beds)	Experimental Group (30 s) (n = 54 Nail Beds)	*p*-Value
	Mean ± SD (95% CI) Median (IR)	Mean ± SD (95% CI) Median (IR)	
Healing time (days)	22.1 ± 3.2 (21.24–22.96) 22 (4)	14.9 ± 2.8 (14.42–15.38) 14.5 (4)	<0.001 **
Recurrence rate	1 (1.8%) ***	2 (3.6%) ***	0.99 ****
* Bleeding (Mild = 1; moderate = 2; abundant = 3)	1.6 ± 0.6 (1.6–1.84) 2 (1)	1.7 ± 0.5 (1.50–1.90) 2 (1)	0.59 **
* Pain at 1st day post-op	1.7 ± 1.6 (1.07–2.33) 1 (3)	1.9 ± 1.8 (1.19–2.61) 1 (2)	0.65 **
* Pain at 2nd day post-op	1.1 ± 1.3 (0.59–1.61) 1 (2)	1.2 ± 1.3 (0.69–1.71) 1 (2)	0.72 **
* Pain at 3rd day post-op	0.7 ± 1.1 (0.26–1.14) 0 (1)	0.8 ± 1.1 (0.36–1.24) 0 (0)	0.79 **
* Post-op inflammation (circumference in cm)	0.3 ± 0.3 (0.18–0.42) 0.2 (2)	0.2 ± 0.2 (0.12–0.28) 0.1 (1)	0.47 **
Post-op Infection	1 (1.8%) ***	1 (1.8%) ***	0.48 ****

Abbreviations: CI, confidence interval; IR, interquartile range; SD, standard deviation; VAS, visual analogue scale. * Bleeding, pain and inflammation indicators have been evaluated according to the affecting toe ** Mann–Whitney U test. Statistically significant differences were set at *p* < 0.05 with a 95% CI. *** Qualitative variable; frequencies (percentage). **** Yates’ Chi- Square test.

**Table 2:** Studies of the Phenol Chemical Matricectomy.

Studies	N (nails)	Phenol Application Time (minutes)	Phenol (%)	Healing Time (days)	Follow-Up (months)	Recurrence Rate (%)
Morkane et al.	54	3	80	-	14	7
Van der Ham et al.	125	3	-	15	19	9.6
Buckley et al.	201	-	80	-	> 6	4.5
Bostanci et al.	350	-	-	14–28	17	0.6
Kordiak et al.	156	8–10	90	14–28	-	2.5
Hassel et al.	112	2	90	7	10	31.5
Vaccari et al.	197	1	88	14–28	24–36	1.5
Karaka and Dereli	348	2	88	14–28	24	0.3
Taticlan et al.	148	1, 2, 3	88	14–17	24	12.5–3.1
Muriel-Sánchez et al.	36	1	88	21	> 6	2.8
Our data (Control Group)	27	1	88	22	> 6	1.8
Our data (Experimental Group)	27	0.5	88	15	> 6	3.6

## Data Availability

Please contact pmj@us.es with any data requests.
